# The Medico-Social Aspects of the Consumption Problem

**Published:** 1913-05-10

**Authors:** 


### THE MEDICO-SOCIAL ASPECTS OF THE CONSUMPTION PROBLEM.

### III.—Consumption Cures in Relation to Treatment.

There are certain consumptive patients who will not persist with the ordinary hygienic treatment, and fly from one variety of advertised cure to another. Most medical men are perfectly well aware that the great majority of these advertised cures and "special methods" of treatment are farcical impostures which owe their primary popularity to the fact that by their means cases of bronchitis and other lung conditions have been much benefited, the patients in such cases thinking that they have been cured of consumption. We have already referred to the difficulties that are met with in diagnosing pulmonary consumption, especially in children. Moreover, every doctor knows that there are cases in which a diagnosis of phthisis is warranted by the facts and signs and yet appear, in some instances, to recover spontaneously under the simplest treatment.

### Natural or Acquired Immunity.

It is difficult to explain these cases, but the most probable cause of their aberrant course is the natural or acquired immunity of the patient, whose vital energies are sufficient to prevent the spread of the disease. It is well known that bacterial invasion of the tissues causes, synchronously with the elimination of the bacterial toxins, the elimination of a bacterial antitoxin which to some extent counteracts the toxins excreted by the organisms. To deal with the whole question here would take us into the debatable field of immunity and its interesting problems, but it is worth while to refer to two recent views which have received acceptance from many medical men. One of these is with regard to diphtheria carriers. It is held by some experienced doctors that such carriers are in a measure safeguards to the community instead of being dangers, since they allow of contacts to be exposed to a low type of infection and to acquire immunity almost with the surety of persons who are inoculated. This view finds strong support in the figures quoted with regard to diphtheria carriers in public schools. The other view is that those who are exposed to tuberculosis and who have the bacillus in their bronchial tubes, by swallowing their phlegm unconsciously produce a state of auto-inoculation which is a similar safeguard. These suggestions will enable us to realise how difficult it is in any given case of incipient pulmonary tuberculosis to make an accurate prognosis since there are so many and such constantly varying factors to be reckoned with.

We point out these facts to emphasise the point that in many cases the nostrums advertised as sure cures for consumption undoubtedly do benefit the patient. Such benefit is not indirect as we might expect, but in some cases direct owing to the fact that many of these decoctions and mixtures contain soothing drugs which act upon the bronchial mucosa and relieve spasm, irritation, and inflammation. It is needless to say that none of them contains a specific against the disease, since no such specific has as yet been discovered. The claims made on their behalf are in most cases so grossly exaggerated that any person of common sense can discount them. Unfortunately, the consumptive patient, especially one in the later stages of the disease, is singularly optimistic and credulous, and he readily accepts what the advertisements tell him as proved facts. That being the case, the medical attendant can only counsel the patient to give both the faddist cure and the hygienic measures a chance. At a sanatorium, usually, such extra medical treatment is interdicted, and quite rightly so. But in private practice little harm will be done by allowing the patient to take an innocuous mixture which is " warranted to cure consumption." The taking of this alleged medicine is a psychological stimulus to the patient, and when aided by proper open-air treatment such suggestive stimulus may do good.

### Findings in " Secret Remedies."

Although there are at present no reliable analyses of these cures, the presence of harmful alkaloids, such as opium and its derivatives, can be ascertained, and the findings given in the B.M.A.'s little book on " Secret Remedies " will prove of some help to the physician in determining how far he is justified in allowing his patient to continue the use of such a remedy.

A different class of cure is found in systems which rely not on medicine, but on exercise, hygienic measures, and especially upon strengthening the respiratory muscles and " hardening " the bronchial system by the use of special diets. One of these systems has had an immense popularity and has been extensively discussed in the professional and lay press. While it can safely be asserted that this system is no cure for consumption, it must be admitted that the measures on which it relies are of the utmost value in the treatment of the disease. Deep breathing is of the greatest value in certain cases, but it must be used and advised with discrimination. The medical man is best fitted to supervise such exercises and to guard against the patient's overtaxing his strength, or promoting instead of retarding the course of the disease by straining his pulmonary muscles.

The Dietetic Treatment.

Similarly with regard to diet. Medical men might usefully devote more attention to this part of the treatment of consumption, and investigate the effects of different diets upon the course of the disease. Some investigation has already been carried out, and the results tend to show that in certain cases a salt-free diet is an admirable adjuvant in the treatment of consumption by hygienic measures. At present, most of us are grouping certain cases into definite classes, in one of which diet treatment is undoubtedly of benefit, while in the others it seems to have no effect whatever upon the disease. The influence of certain diets upon cases of chronic bronchitis with marked bronchiectasis has been reported upon by German investigators, and their findings are sufficiently encouraging to stimulate others to further research. All we can safely say at present is that by diet alone, no case of consumption, definitely established, can be cured, but that with proper out-door treatment a specially regulated diet is a useful aid which should never be neglected, but should in all cases be supervised by the doctor.

